# A conceptual model and practical guidance for the development, administration, and evaluation of individualized therapies

**DOI:** 10.3389/fmed.2025.1493832

**Published:** 2025-01-24

**Authors:** Lucie Perillat, Andrew McFadyen, Patricia Furlong, James Anderson

**Affiliations:** ^1^Department of Molecular Genetics, University of Toronto, Toronto, ON, Canada; ^2^Program in Genetics and Genome Biology, The Hospital for Sick Children, Toronto, ON, Canada; ^3^Precision Child Health, The Hospital for Sick Children, Toronto, ON, Canada; ^4^Division of Clinical Public Health, University of Toronto, Toronto, ON, Canada; ^5^Department of Bioethics, The Hospital for Sick Children, Toronto, ON, Canada; ^6^Parent Project Muscular Dystrophy, Washington, DC, United States; ^7^Institute of Health Policy, Management, and Evaluation, University of Toronto, Toronto, ON, Canada; ^8^AI at SickKids, The Hospital for Sick Children, Toronto, ON, Canada

**Keywords:** ethical challenges, regulatory implications, CRISPR/Cas9 therapeutics, research-care dichotomy, bespoke therapy, Nof1, rare diseases

## Abstract

Bespoke therapies represent a promising tool to address a diverse range of genetic and acquired conditions, offering new hope where conventional treatments have fallen short. With the rapid rise of bespoke therapies, profound ethical and regulatory challenges emerge, making it crucial to establish a comprehensive framework that ensures these treatments reach clinical settings and meet patients’ needs as quickly as possible while protecting all parties involved. Although current guidelines are continually evolving to address the range of ethical tensions raised by these therapies, several gaps remain. A significant unresolved question is determining where personalized interventions fall on the research-care continuum and understanding the institutional, regulatory, and ethical implications when custom therapies are classified as research, care, or a mix of both. To address these questions, we introduce a conceptual model alongside practical guidance for the development, administration, and evaluation of individualized therapies, using CRISPR/Cas9-based interventions for Duchenne Muscular Dystrophy as a case study. We argue that the goals of an intervention should be as individualized as the bespoke product itself, tailored to the specifics of each case. Rather than attempting to pinpoint the exact location of an intervention on the continuum, which may be hard to operationalize and have limited utility, our approach focuses on the practical details of how such interventions are administered and the individual component parts of an intervention. It advocates for transparent discussions among all partners to anticipate and adjust various components/parameters along the process of administering individualized interventions. Our paper highlights the most critical of these parameters in (1) the planning and development of individualized therapies in laboratory settings, (2) their regulatory oversight, and (3) evaluation. By discussing these stages and parameters in detail, we aim to provide guidance on how to navigate the ethical complexities inherent to individualized interventions and offer a preliminary framework for balancing the interplay between research objectives and patient care needs. Acknowledging that the scientific rigor and adequacy of any new model must be evaluated, we also identify the types of evidence that are required to validate that our model effectively meets individual and societal needs.

## 1 Introduction

Bespoke, or individualized, therapies are quickly emerging as an increasingly valuable tool to address a myriad of genetic or acquired conditions, providing hope to patients who previously lacked any promising treatment options. This rapidly expanding branch of research and medicine includes developing and improving tools such as gene and cell therapies, genome editing and modulation strategies (e.g., CRISPR/Cas9, antisense oligonucleotides), personalized immunotherapies (e.g., CAR-T cells), and stem cell therapies. To date, 38 gene and cell therapies have been approved by the FDA (1), the most recent of which is Casgevy, a CRISPR/Cas9-based *ex vivo* therapy for sickle cell disease and beta thalassaemia (2). Since 2020, the FDA has received more than 200 investigational new drug (IND) applications for cell and gene therapies annually and expects to approve 10–20 new products per year by 2025 (3, 4). While this renaissance will have a profound impact on human health, it also brings a growing tension between the need to advance such ground-breaking, and potentially curative, treatments, and the ethical challenges associated with individualized, “one and done” therapies.

The novelty associated with these emerging technologies raises unique challenges, both technical and ethical. Historical instances like Jesse Gelsinger’s death from a gene therapy trial for ornithine transcarbamylase deficiency (OTCD) in 1999, and leukemia cases from insertional mutagenesis in the SCID-X1 trial, demonstrate that risks are complex and arise not just from the technology but also the ecosystems of its development, approval, and administration (5). When it comes to clinical trials for individualized therapies, shortcomings in regulatory and ethical oversight might have a profound impact not just on the patient involved, but also on current and future patient populations. Jesse Gelsinger’s death, for example, is often cited as a setback that may have delayed progress in the field of gene therapy by a decade (5, 67). Nevertheless, it also spurred substantial changes in the way clinical trials are conducted and regulated. As personalized therapies are becoming increasingly common, it is essential to develop a robust framework to facilitate their translation to the clinic at an appropriate speed, while ensuring the protection of all parties involved - patients and families, scientists, clinicians and institutions (6).

Although current guidelines are continually evolving to address the wide range of ethical tensions raised by these therapies, several gaps involving equity of access, informed consent, and risk/benefit calculations remain (68). Additionally, the administration of individualized therapies may involve varying degrees of clinical research and clinical care, which are often intertwined and exist on a continuum, as described by Crowden et al. (7).^1^ A key unresolved issue is determining where personalized interventions fall on the research-care continuum and understanding the institutional, regulatory and ethical implications when custom therapies are classified as research, care, or a mix of both. Considering that the term ‘n-of-1 trial’ suggests a research focus while ‘individualized therapy’ implies a therapeutic goal—and acknowledging the ambiguity that persists between these concepts—we will use the term ‘individualized intervention’ as a more neutral alternative in this paper.

To address these questions, we introduce a conceptual model alongside practical guidance for the development, administration, and evaluation of individualized interventions, with a particular focus on CRISPR/Cas9-based interventions, using Duchenne Muscular Dystrophy as a case study. We argue that the goals of an intervention should be as individualized as the bespoke product itself, tailored to the specifics of each case. In our model, each intervention is conceptualized as being made up of individual components, or parameters, which can be adjusted to either enhance societal benefits (i.e., research) or benefit the individual patient (i.e., care). Rather than attempting to pinpoint the exact position of an intervention on this continuum, which may be hard to operationalize and have limited utility, our approach encourages open discussions among all parties involved, hereafter called partners, to anticipate and adjust the different practical parameters that are encountered along the process of administering individualized interventions. In our new model, these various parameters are tailored to the specifics of each case and evaluated separately from a research or care perspective. This paper highlights the most critical of these parameters in (1) the planning and development of individualized therapeutics in laboratory settings, (2) their regulatory oversight, and (3) post-intervention evaluation. By discussing these stages and parameters in detail, we aim to provide guidance on how to navigate the ethical complexities inherent to individualized interventions and offer a preliminary framework for understanding and balancing the interplay between research objectives and patient care needs. Acknowledging that the scientific rigor and adequacy of any new model must be evaluated, we also identify the types of evidence that are required to validate that our model effectively meets individual and societal needs.

## 2 Navigating the research-care continuum: the theory

Although “trials of therapy,” in which clinicians test certain treatments and evaluate an individual’s response to them, have been routinely used in medical practice, the term “n-of-1 trial” was officially coined in 1986 by a group of clinicians at McMaster University (Canada) following a study on the use of theophylline in a patient with poorly controlled asthma^2^ (8). Since then, n-of-1 trials have typically taken the form of “crossover” trials, where a patient follows a sequence of two or more treatment periods – alternating between the intervention and a comparator - in a controlled, blinded and randomized manner (9, 10). This type of design has been used in different fields, from oncology (11) to physiotherapy (12), with growing uncertainty around whether they should be classified as research or care.

This issue is exacerbated for CRISPR-based as they cannot follow a traditional crossover research design. Crossover trials are considered appropriate only if the illness remains stable throughout the evaluation period (e.g., chronic conditions), and if treatments exhibit rapid and dramatic “on/off” effects and have short wash out periods (9, 13, 14). CRISPR-based interventions, as well as gene replacement therapies and genome editing strategies, do not fulfill these requirements as their effects may be permanent and irreversible and, therefore, require a different type of trial design. “Pre-post” trials, in which a patient’s outcomes are compared before and after treatment, offer such an alternative (10, 15). These trials have been described as “prospectively designed case reports of innovative care,” given the absence of research features such as randomization or blinding (10), p. 1679.

Several concerns have been raised regarding n-of-1 trials, including the difficulty of maintaining a rigorous research design, performing robust statistical analyses, and ensuring external validity (9, 10). As Kane et al. (10), concerns surrounding traditional (crossover) n-of-1 trials are exacerbated in pre-post trials due to the even less established standards for their design and reporting.

Compounding these concerns, the administration of individualized therapeutics can theoretically fall under several classifications along the research-care continuum, raising further ethical and practical challenges. On the one hand, the administration of bespoke therapies can be viewed as an act of innovative practice or medical care (16), guided by clinical ethics principles, and enabled through discussions between patient families, clinicians and bioethicists around risks and benefits of the intervention. On the other hand, it can be considered a formal research activity, bound by the norms of clinical research, research ethics and regulation. Finally, some interventions may be administered under pathways such as off-label use or compassionate use/expanded access and Right to Try, some of which encourage data collection to inform future research and care efforts^3^ (17, 18), thereby incorporating elements of both research and care. Amid these categorizations, a central question arises: How do the distinct goals of research and care intersect with the administration of individualized interventions?

To begin to answer this question, we will briefly review the existing literature to delineate the respective goals of the research and care enterprises. The primary goal of medicine revolves around the provision of care, which includes diagnosing, alleviating or curing illnesses to maintain or restore health. This encompasses preventing the onset of diseases and minimizing their impact once they arise to improve or maintain a certain quality of life (19–21). Care is often flexible and dynamic and intends to benefit the individual patient, following ethical principles such as beneficence, non-maleficence, autonomy and justice (22). In the context of care, clinicians face the complex task of using population-level data to make inferences about the individual patient, which Montgomery refers to as “*particularization*” (23, 24).

In contrast, the research enterprise operates on a different dynamic where researchers aim to determine whether findings observed in specific cases (the study population) can be *generalized* beyond the study population, thereby providing societal benefits (25). To accomplish this feat, research adheres to strict inclusion and exclusion criteria, and rigid study protocols (26). Since these processes may pose risks to research participants, research is also subject to ethics oversight and regulation.

Having established the overarching goals of research and care, we now examine existing arguments that aim to categorize the administration of individualized interventions as either a research endeavor or a component of clinical care. A segment of the existing literature advocates for individualized interventions to be administered under a therapeutic warrant, thereby prioritizing direct benefits to the individual patient and, usually, faster access to the intervention due to a lower regulatory burden. Such arguments typically revolve around the presupposed inability of n-of-1 trials to produce generalizable knowledge (which is considered a cornerstone of the research endeavor) due to poor internal and external validity (10). As mentioned above, CRISPR-based interventions and other genome editing strategies cannot follow a traditional crossover design and may, therefore, lack control groups, randomization and blinding, three central features of what is widely considered the gold standard of research evidence (27). Another feature deemed essential to research activities, dating back to Aristotle and more recently emphasized in frequentist statistics, is the need for repeated experimentation and data collection over time to make reliable scientific inferences (69). This iterative process is, by definition, absent from n-of-1 trials, which may seem to give further weight to the argument that individualized interventions should be exclusively viewed as a part of clinical care.

Alternatively, another segment of the literature has argued that the administration of individualized interventions should be categorized as research. For instance, Kimmelman contends that the risks associated with gene therapy first-in-human trials are justified solely by their potential societal benefits and should not be labeled as therapeutic (5, ch. 10). Others supporting the categorization of individualized interventions as research have emphasized the generalizability of n-of-1 trials. Notably, it has been shown that 60% of published n-of-1 trials are aggregated as part of a series (i.e., a single report that publishes n-of-1 trial data from multiple participants receiving the same condition-specific intervention) (8, 28), thereby facilitating comparisons across studies and generating valuable insights that can benefit patients beyond the original trials (29).

Discussions around the generalizability of n-of-1 trials have typically, at least until very recently, revolved around a rather constrained conceptualization of external validity. In these discussions, the focus is typically on whether a specific *product* can be used in a particular patient group beyond the study itself (30). This limited view of external validity led to the assertion that n-of-1 trials, which aim to test a product that is specifically tailored to a single patient, lack generalizability.

However, we, among others, believe that embracing a broader view of generalizability may be helpful in revealing the potential benefits of n-of-1 trials for diverse patient populations. Below, we outline how, using a broader understanding of generalizability, n-of-1 trials allow us to (1) evaluate whether a comprehensive treatment algorithm, not just the medical product itself, can be applied to diverse patient populations, and (2) gain mechanistic and causal insights that can help stratify patient populations and test broader scientific theories.

First, n-of-1 trials can serve to evaluate comprehensive treatment approaches or algorithms, also known as “intervention ensembles” (30, 31). These ensembles include not only the therapeutic substance itself, but also co-interventions, dosing schedules, diagnostic methods, and constraints specific to different treatment settings. There are endless possibilities for integrating a particular product into various intervention ensembles, yet only a few, if any, will demonstrate efficacy and gain approval.

The concept of intervention ensembles underscores the necessity of testing specific combinations of factors whose effectiveness may vary depending on contextual variables. This parallels the sensitivity to local variations seen in other fields, such as Artificial Intelligence, where what succeeds in one setting may not in another, emphasizing the importance of assessing the architecture of the ensemble rather than focusing solely on outcomes. By leveraging the concept of intervention ensembles, we show that n-of-1 trials can indeed be generalizable: while the medical product itself may be tailored to an individual patient, and thus not be generalizable, other components of the intervention ensemble may have broader applicability (10, 32, 33). For example, Milasen, an antisense oligonucleotide (ASO) developed for a patient with an ultra-rare disorder, was developed to treat a single patient, yet the ASO technology and the general intervention platform is being studied for other conditions (32, 34).

Even more broadly, we argue that n-of-1 trials, if appropriately designed, can contribute to generating knowledge applicable to future (and diverse) patient cohorts. Mechanistic and causal insights gained from individualized studies can guide management of patients with similar or related diseases or those planning to undergo interventions using similar technologies. Due to the intensive monitoring of patients typical of n-of-1 studies, patient response can easily be associated with detailed clinical and demographic data (35). By identifying commonalities across multiple or aggregated n-of-1 studies (36), researchers can distinguish between patients who respond positively to a type of intervention from those who do not. This, in turn, may allow for the stratification of patients, enhancing the quality of patient care (37). Stepping back even further, well-designed n-of-1 trials can yield insights not only into the effects of the intervention ensemble but also into broader theories guiding their development and intended application. Such insights might facilitate the generation of novel hypotheses about disease mechanisms, therapeutic actions, and interactions with co-interventions.

Circling back to the respective goals of research and care, some scholars have argued that both research and medicine are fundamentally driven by the shared goal that is the pursuit of knowledge (19). In medicine, this manifests in an effort to diagnose, understand the causes of diseases, predict outcomes, and identify effective treatments for patients. In contrast, research seeks to advance knowledge, either for its intrinsic value or for practical applications in fields such as medicine and technology. Importantly, the distinction between research and clinical care is sometimes blurred, and, as Kimmelman notes, their interaction does not always follow a straightforward path from laboratory discoveries to bedside applications, as can be implied by the expression “from bench to bedside” (5, ch. 6). One could argue that incorporating aspects of both the research and therapeutic undertakings into the administration of individualized interventions would fulfill this shared goal of ensuring timely access to the intervention and benefits to the individual patient, while also facilitating the collection of valuable data that may be beneficial to diverse patient populations in the future.

Recently, and in alignment with the argument outlined above, individualized interventions have been described as a ‘research-treatment hybrid’ (32). As mentioned by the authors, this concept is descriptive and not entirely novel, as the administration of individualized interventions has sometimes already intertwined the goals of generating generalizable knowledge and benefiting the individual patient (32). Although the ‘hybrid’ terminology opens the door to a new model for the administration of individualized interventions, it perpetuates the high-level dichotomy between research and care.

## 3 Individualized interventions: a new conceptual model

In this paper, we argue that there is no universal answer to where individualized interventions generally fall on the research-care continuum: some interventions will primarily aim to benefit the individual patient (i.e., care), while other will tend toward also generating generalizable knowledge or enhancing societal benefits (i.e., research). Of important note, these options lie on a continuum, with numerous possibilities beyond the two outlined here.

**Figure 1:**
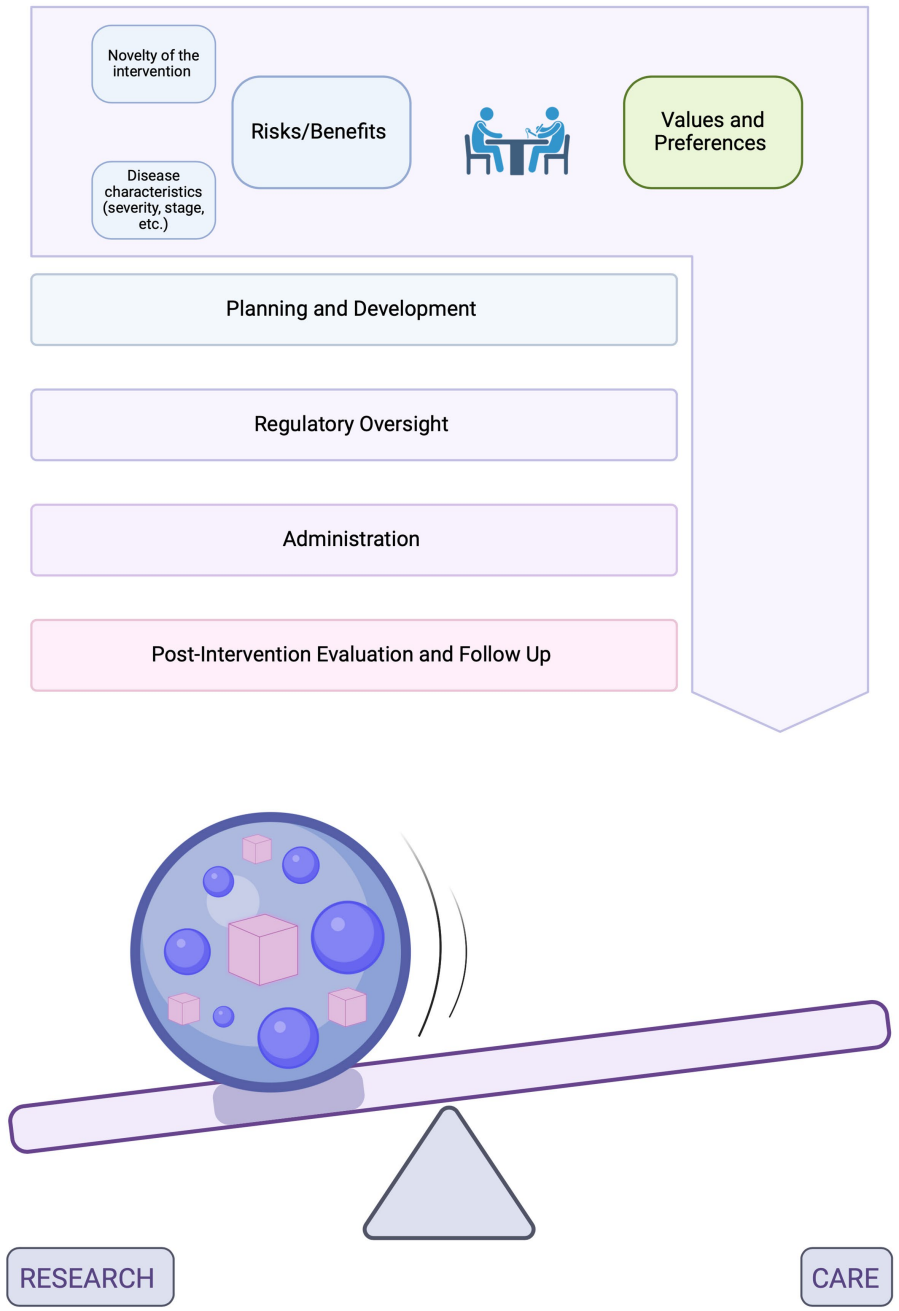
A conceptual model for the administration of individualized interventions. The top diagram represents the process spanning the planning and development of individualized interventions in laboratory settings, their regulatory oversight, administration to patients and evaluation post-intervention. In the bottom diagram, the sphere represents this entire process. Inside the sphere are different parameters which can be adjusted based on the overarching goals of the intervention (previously established through discussions among all partners). These parameters are found within each of the steps of the process and may include, for example, the amount and type of data collected during trials or the extent of data sharing and reporting, among others. Some of the most important parameters will be discussed in further details in the sections below. Parameters that are adjusted to generate generalizable knowledge or enhance societal benefits (i.e., research) are represented by purple spheres and parameters that are adjusted to benefit the individual patient (i.e., care) are represented by pink cubes. The weight and combination of these components, which would be unique to each patient and intervention, would help estimate where the intervention falls on the research care continuum. The presence of the ball represents a range of such possibilities (not an exact point on the continuum) and indicates that this categorization can be dynamic [Created in BioRender. Perillat (2024) BioRender.com/d59g629].

This supposition leads us to argue that the goals of an intervention should be as individualized as the bespoke product itself, tailored to the specifics of each case. In our model, each intervention is conceptualized as being made up of individual components, or parameters, such as the amount and type of data collected during trials or the extent of data sharing and reporting, among others. Individual parameters can be adjusted to either generate generalizable knowledge or enhance societal benefits (i.e., research) or to benefit the individual patient (i.e., care). Rather than attempting to pinpoint the exact position of an n-of-1 trial on this continuum, which may be hard to operationalize and have limited utility, our approach encourages open discussions among all parties involved to anticipate and adjust the different practical parameters that are encountered along the process of developing, administering, evaluating, and monitoring individualized interventions (Figure 1). In our new model, these various parameters are tailored to the specifics of each case and evaluated separately from a research or care perspective. As a result, both sets of ethics principles (research and therapeutic) can effectively be applied, rather than forcing a choice between them^4^. As seen in Figure 1, the weight and combination of these individual components would help *estimate* where the intervention falls on the research-care continuum.

This approach is based on the supposition that the research-care distinction has limited utility when applied at a higher (i.e., whole intervention) level, but that it can, and should, do important work when assessing the individual component parts of a trial. Our model, therefore, focuses on the practical details of how such interventions are developed, administered, evaluated, and monitored.

The following sections will provide an operational framework and specific recommendations regarding how to discuss and adjust these practical parameters in (1) the development of individualized interventions in laboratory settings, (2) their regulatory oversight, and (3) post-intervention evaluation. By focusing on the individual component parts of the intervention, our model hopes to be more granular than the broader, yet useful concept of a ‘research-treatment hybrid’ (32).

## 4 Case study: CRISPR/Cas9 individualized interventions for Duchenne Muscular Dystrophy

To clarify the arguments presented here, we will use the case study of the development of CRISPR/Cas9 based personalized treatments for Duchenne Muscular Dystrophy (DMD) patients. DMD is a rare X-linked disorder caused by mutations in the *DMD* gene that result in the malfunction or loss of the dystrophin protein required for muscle contraction and integrity (38). DMD patients present with muscle weakness which leads to a loss of ambulation in their teen years, eventually followed by fatal cardiorespiratory failure in their 20s or 30s. While glucocorticoids - the current standard of care for DMD patients - slow progression of the disease, they only manage the symptoms of the disease and do not address its root cause (39).

The advances made in the field of genome editing, and the use of CRISPR/Cas9 specifically, allow for the modeling and successful treatment of genetic disorders (2, 40). CRISPR/Cas9 is a programmable gene editing tool comprising an endonuclease protein, Cas9, that cuts strands of DNA, and a single guide RNA (sgRNA) that directs where Cas9 cuts (41). The power of the CRISPR/Cas9 technology can be harnessed to both model different types of mutations and correct pathogenic mutations (42, 43), thereby leading, in the case of DMD, to the potential restoration of a full-length dystrophin protein. Importantly, such gene editing strategies show promise to stop the progression of the disease but not reverse symptoms. In other words, for DMD and many other genetic conditions, once damages occur, they cannot be reversed.

Gene therapy and CRISPR/Cas9-based interventions present a myriad of unique challenges arising from their complex nature and mechanisms of action. In both cases, the system contains a mixture of several components, such as the transgene, promoters, capsid genes, each potentially introducing risks, including the possibility of horizontal or vertical gene transfer. Moreover, concerns exist around insertional mutagenesis (or off-target effects for CRISPR/Cas9 systems) in tumor suppressing genes or oncogenes potentially leading to the development of cancers (5, 68, ch.2).

These important risks aside, arguably the most significant concern around these approaches is the very real possibility of triggering strong immune reactions against the vector, transgene or even the Cas9 protein (44, 45, 68). In the context of DMD, CRISPR/Cas9-based interventions are typically delivered to muscle tissues using viral vectors, which have been linked to strong immune reactions and hepatotoxicity (45). Traditionally, the AAV9 vector has been used for DMD but the field is now moving toward novel, possibly more efficient and less immunogenic vectors, such as MyoAAV (46). MyoAAVs are vectors that have been shown to preferentially target muscle tissues and de-target the liver in animal models, thereby enhancing efficacy while reducing immunogenicity (47). Their increased efficacy may allow for lower doses, therefore decreasing the risk of triggering strong immune reactions. While promising results have been observed in animal models, the efficacy and safety profiles of MyoAAVs are yet to be determined in humans.

The issue of immunogenicity, which plagued the OTCD trial and led to Gelsinger’s death in 1999 (5), is compounded by the fact that there exist significant disparities in immune reactions between animal models and humans (48). The occurrence of immune responses directed against the capsid raises questions regarding the eligibility of trial participants in current and future trials (49). Finally, due to the rapidly changing nature of the field, certain features of the system may be quickly replaced by others, such as the replacement of AAV9 by MyoAAV, which sometimes complicates the evaluation of risks and benefits of the proposed intervention (5, ch. 4).

In the context of DMD, we will use three different scenarios: (1) Family A, with a 4-year-old patient planning to receive a CRISPR/Cas9 personalized treatment packaged in the traditional AAV9 vector, (2) Family B, with a 10-year-old patient planning to receive a CRISPR/Cas9 treatment packaged in AAV9, and (3) Family C, with a 10-year-old patient planning to receive a CRISPR/Cas9 treatment packaged in the new MyoAAV vector. We acknowledge that, although helpful to illustrate our model, these three scenarios do not represent the diversity of patient populations (age, severity), delivery method/vector, therapeutic system (e.g., CRISPR editing, gene replacement, promoter).

## 5 Navigating the research-care continuum: practical considerations

### 5.1 Step 1: initial discussions around overarching goals of the intervention

In our model, transparent discussions among all parties involved are essential to facilitate an initial agreement regarding the overarching goals of the intervention and, subsequently, guide decisions regarding various parameters along the process of developing, administering and evaluating individualized interventions. For these decisions to be made, two key aspects must be discussed: (1) the values and preferences of all parties involved and (2) the risks and benefits of the intervention, including its novelty, and characteristics of the disease, such as severity and stage. We will address these two points in turn below. As shown in Figure 1, although it is essential to start these discussions before therapy development begins, they will likely need to be revisited as circumstances evolve and new decisions arise.

First, discussions around values and preferences of all partners, including patient and families but also researchers and clinicians, would help identify and clarify priorities, such as whether the primary/only goal of the intervention is to benefit the individual patient or whether it may be feasible and desirable to also evaluate the generalizability of an intervention ensemble with the aim of benefiting future patient populations.

This initial decision may be influenced by several tangible factors, including the novelty of the intervention: as Kimmelman proposes, more novel interventions (that are likely to carry greater risks) may be more justifiable based on societal benefits (5, ch. 5). He claims that novel interventions would include those that “involve one of the first attempts to target a particular biological pathway, one of the first uses of a new vector, one of the first uses of a new transgene, one of the first attempts to target a particular tissue, or one of the first attempts to use a new platform against a disease (e.g., the first gene-transfer trial against multiple sclerosis)” (5), p. 80.

The initial decision regarding the overarching goals of the intervention might also be shaped by characteristics of the disease: for example, a patient in the later stage of a rapidly progressing disease might want to prioritize rapid access to the intervention, thereby benefiting the individual patient, instead of the generation of generalizable data, benefiting society at large (50).

Using the three scenarios mentioned above, several factors emerge that should be carefully considered at this stage. While all three families might naturally want to prioritize the well-being of their child, various factors could influence the extent to which resources and time may also be allocated toward generating generalizable knowledge and, possibly, enhancing societal benefits. While it may not be feasible or necessary to precisely quantify this, it is valuable to gage the preferences of all partners at this stage. For instance, in the case of Family A, whose child is 4 years old, the urgency of accessing the intervention might not be as pressing as it is for Family B, whose 10-year-old child has likely already experienced significant muscle damage and may be approaching the end of the therapeutic window. However, one could also argue that Patient A might benefit more from the intervention than Patient B, as most of their muscle tissue remains undamaged, and may also better tolerate moderate side effects, thereby reducing associated risks. Meanwhile, Patient C is the same age as Patient B but will receive a vector that has never been used in humans before (MyoAAV). The safety and efficacy profiles of MyoAAV, as well as the minimum dose required for efficacy, remain uncertain in humans. According to Kimmelman, novel interventions such as this one should be justified based on societal benefits (5, ch. 5), which means that the design of the intervention (and clinical endpoints) may need to be adjusted to maximize the generation of new knowledge that could benefit future patients treated with MyoAAVs.^5^

Of important note, after selecting a disease for the development of an individualized intervention, patient selection within the affected population should also address justice concerns. For example, prioritizing patients in earlier disease stages may increase clinical success rates, but this approach might disadvantage individuals with more advanced disease stages who have limited research participation opportunities. The question of how institutions should handle equitable access to individualized interventions through n-of-1 trials is a complex question that will be discussed in a later section of this paper and a follow up paper.

The above examples provide just a glimpse into the diverse perspectives that can be considered in each of these cases, highlighting the crucial need for early and transparent discussions among all parties involved to ensure that patient-specific factors, partners’ preferences as well as risks associated with the intervention are considered. As demonstrated in the example above, each case is inherently complex, and while initial discussions around the intervention’s goals are crucial, they should not become an overly burdensome or time-consuming process, further delaying patient access to therapy. In these discussions, it is essential to strike a balance between the inputs provided by the patient/family and those provided by the professionals and institutions facilitating the trial. While family values and preferences may guide some of the decision-making, certain aspects must also be informed by more objective or tangible considerations related to evidence, novelty, risk and commensurate requirements for benefit at a societal level (knowledge). Given that the involved parties may hold diverse, and sometimes conflicting, perspectives that could take considerable time to reconcile, it may be beneficial to involve an independent board of bioethicists and patient advocates to moderate and facilitate these discussions. Further work is required to determine the relative weight of each partner’s input in the decision process.

By emphasizing the importance of early, transparent discussions among all parties involved, our model helps in setting clear and transparent priorities and expectations from the outset, ensuring that all perspectives are considered and integrated into the decision-making process, thereby minimizing therapeutic misconception and overestimation. Therapeutic misconception arises when study participants do not differentiate between the objectives of clinical care and clinical research. This confusion can lead patients and families to overestimate the benefits of study participation which, in turn, can lead them to not fully weigh the potential risks, limitations, or purpose of the intervention, thereby negatively impacting their ability to provide true informed consent.^6^

Additionally, in the case of bespoke interventions, family members frequently assume a multi-faceted role, acting not only as caregivers and consent providers but as financial sponsors, project managers, and co-designers of the study. They might have dedicated numerous hours to fundraising for the trial, benefited from a successful crowdfunding or public campaign, or possibly both. They could have established connections with medical institutions or practitioners, presenting the concept of personalized interventions along with the commitment of financial resources to support the project’s progress. As such, the lines between patient/decision-maker and sponsor are often blurred. Caregivers may become so deeply involved in the process that they struggle to objectively evaluate the risks and benefits associated with administering the intervention, further jeopardizing the informed consent process.

The above considerations underscore the need to thoroughly discuss the intervention’s objectives, partners’ preferences, and associated risks. This process is crucial for fostering transparency in decision-making, setting realistic and clear goals, and minimizing therapeutic misconception and overestimation. Once the overarching goals of the intervention are defined, further discussions can help refine various parameters throughout the development, administration and evaluation of the intervention, ensuring that the approach remains aligned with patient-specific needs and broader objectives. Some of the key parameters that should be adjusted for each patient and intervention are detailed in the following sections.

### 5.2 Step 2: planning and development

When institutions, such as sponsors and research institutes, begin planning for the development of an individualized intervention, they must navigate key decisions that our new model is designed to address with greater flexibility and transparency. Previously, scientists may have encountered roadblocks during the development of individualized interventions due to the ambiguity of whether these interventions should be categorized as research or care. We hope our new model empowers partners to make informed choices regarding two critical parameters in the planning and development process: (1) deciding whether to replicate the specific patient’s circumstances as closely as possible in preclinical studies or, instead, develop a treatment algorithm that, although less patient-specific, may be beneficial to a broader patient population, and (2) deciding whether to allocate resources toward investigating broader questions potentially at the risk of delaying patient access to therapy. We will explore these questions in turn below.

#### 5.2.1 Adjusting translational distance

**Figure 2:**
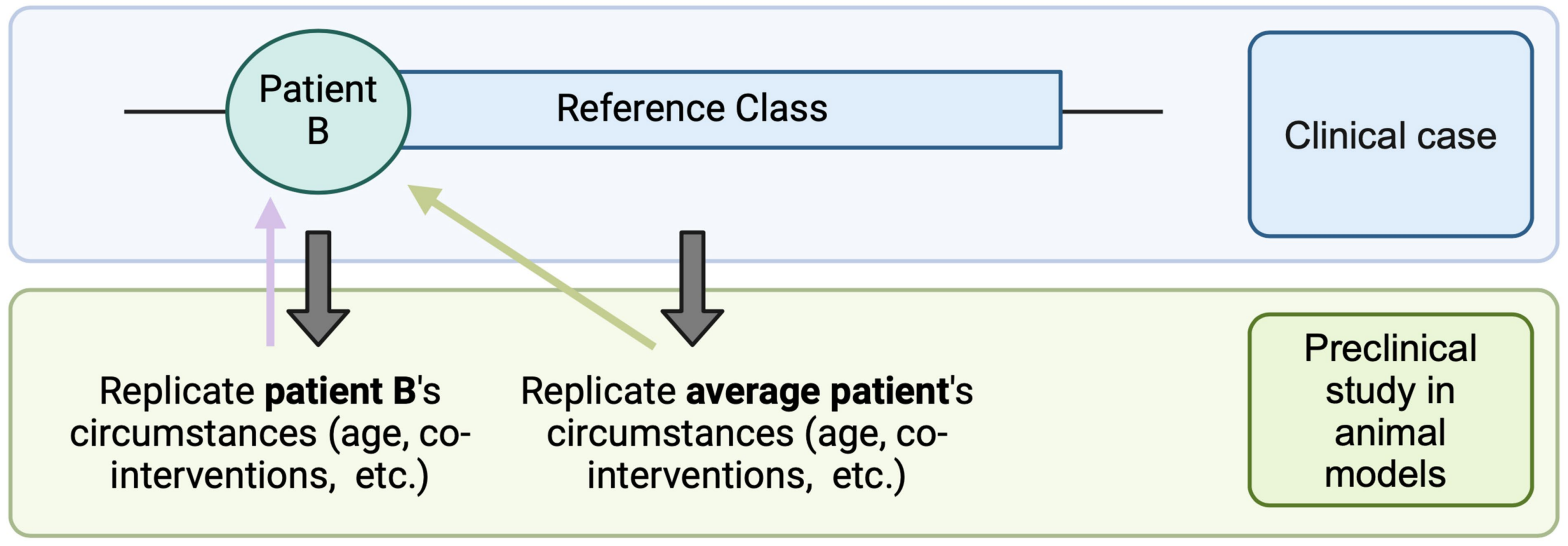
Translational distance. The reference class consists of average cases, which may or may not align exactly with the individual patient’s circumstances. One approach is to replicate the patient’s specific conditions as closely as possible in the preclinical study (e.g., age at intervention, pre-existing treatments). This strategy minimizes the translational distance (purple arrow) between the preclinical study and the administration of the intervention to Patient B in a clinical setting. Alternatively, the preclinical study can replicate the conditions of the average patient (e.g., the optimal age for treatment, standard care practices). While this approach slightly increases the translational distance for Patient B (green arrow), it may offer broader benefits for a larger patient population [Created in BioRender. Perillat (2024) BioRender.com/k61t440].

The first significant challenge researchers may encounter lies in the formulation of the research hypothesis and the development of an appropriate preclinical study design. In the case of rare diseases such as DMD, bespoke therapeutics tend to be tested in an animal model that recapitulates the patient’s genetic mutation. In this context, scientists may need to decide whether to closely replicate the patient’s unique circumstances (genetic, developmental, medical) in the preclinical study to minimize the “translational distance” between preclinical studies and human trials [as notes Kimmelman (5, ch.7), translational distance increases when there exist differences in intervention set up, dosing, immune system of the animal model and the patient, etc.] or, instead, develop a treatment algorithm that may be applicable to a broader patient population (Figure 2). This may involve making decisions about the age/developmental stage at which the animal model is treated: in the case of Patient B, whether it should match the exact age at which the patient would be treated (i.e., early teens) or the age at which most patients should ideally be treated (i.e., right after diagnosis). It might also dictate the types of co-interventions used in the study: for example, whether they should mirror the patient’s current treatments for DMD and for any other pre-existing conditions, or only include the standard of care for DMD, applicable to most DMD patients.

As seen in Figure 2, minimizing translational distance can provide a more accurate estimate of the real-world efficacy of the intervention for the individual patient. However, closely replicating a patient’s unique circumstances, while beneficial for that individual, may limit the intervention’s applicability to a broader patient population.

#### 5.2.2 Resource allocation for investigating broader scientific questions

Another challenge for researchers might be deciding between ensuring rapid patient access to the therapy or dedicating time and resources to investigate questions that may result in broader knowledge acquisition and societal benefits. While both endeavors can be conducted in parallel (and often are), prioritizing one aspect may, in certain cases, come at the expense of the other. Consider, once again, a research team developing personalized CRISPR/Cas9-based interventions for patients with DMD. In this process, they first want to generate animal models for each patient’s mutation, using the same CRISPR/Cas9-based technique but with different sgRNAs tailored to each mutation. They notice that, although the technique is conceptually the same, outcomes (i.e., the likelihood of generating the mutation of interest) vary widely, likely due to interactions between the sgRNAs and specific mutations (considering factors such as mutation location, length, and type). Here, the researcher faces a dilemma: should they investigate the underlying patterns of efficiencies to leverage this knowledge for future model generation, or should they concentrate on advancing the therapy for the current patient through the research pipeline?

Our model encourages early identification of such questions, enabling informed decisions based on prior discussions about preferences and disease stage. For instance, if Family A and all partners involved agree that one of the intervention’s goals is to enhance societal benefits and that time and resources *can* be spent on generating generalizable data, researchers may feel more assured in exploring the underlying patterns behind the varying efficiencies in model generation, even if it might not be of immediate benefit to the patient. Here, we do not seek to evaluate the decision made for Family A, as we believe there is no definitive right or wrong answer. The determination ultimately hinges on a careful assessment of risks and benefits, aligned with the values and preferences of all partners. This approach, however, enhances transparency among parties involved and distributes the responsibility for decision-making across multiple individuals, rather than placing it solely on the scientist or research institute. This, in turn, if adopted more broadly, might help minimize what has been described as “social dimensions of risk management,” which has historically plagued several gene therapy trials (5, ch. 3).

As outlined above, in the planning and development stage, at least two key parameters should therefore be tailored to the specifics of each case: (1) the degree to which translational distance should be minimized to closely mirror the patient’s circumstances in preclinical studies, or, alternatively, broadened to assess the applicability of a more general treatment algorithm, and (2) the balance between accelerating therapy development to ensure timely access for the patient vs. generating novel and more broadly applicable knowledge that could advance the field and benefit future patient populations. As with our conceptual model outlined above, these factors do not represent rigid dichotomies but rather continuums where decisions should be strategically made based on the specific context and goals of the intervention.

### 5.3 Step 3: regulatory oversight

Once a therapeutic product has been developed and evaluated in laboratory settings, regulatory institutions face the critical task of deciding whether to approve it for clinical trial and use. Recent cases have demonstrated that ongoing uncertainty about whether individualized interventions should be categorized as research or care has led to a complex, in some cases, possibly redundant, blend of regulatory oversight processes, encompassing both human subject research and clinical care oversight (32).^7^ The question of which regulatory pathway is most appropriate has already been raised in the context of compassionate use, which also involves varying degrees of clinical research and clinical care (18). Further work is needed to develop a streamlined and possibly less complex regulatory pathway for the approval of individualized interventions, either leveraging existing pathways or developing new ones.

The development of an appropriate regulatory pathway will likely be challenging, as the assessment of risks and benefits associated with individualized interventions is uniquely complex. Research involving children that carries greater than minimal risk must demonstrate direct benefit, unless specific criteria are met, such as the likelihood of generating knowledge that helps understanding (or improving) the child’s condition. Even if this criterion is met, such research can only pose a “minor increase over minimal risk.” By its very nature, an individualized intervention carries a very high level of uncertainty, and it may be impossible to predict direct benefit or associated risks at the time when discussions around informed consent take place. Patients and parents, especially when dealing with a severe disease, may be willing to accept higher risks for the potential of significant benefit, indicating that more than a “minor increase over minimal risk” may be acceptable in the case of patients seeking access to individualized interventions (51). Uncertainty around this issue also raises practical questions, such as whether we should administer lower doses to children to minimize risk, or instead maximize doses to enhance potential benefits, even if this increases the likelihood of dose-related toxicity (50).

These considerations are just one aspect of the assessment made by regulatory institutions, which typically hinges on multiple factors, such as toxicology and pharmacology data derived from preclinical studies, a proposed clinical trial design, and details regarding product preparation and manufacturing (52). Throughout this assessment, several parameters can be adjusted based on the specifics of each case, namely (1) the nature and amount of preclinical evidence required for approval, (2) the clinical trial design, including the amount and type of data collected during the trial, and (3) the extent to which the trial incorporates generalizability and data standardization practices.

#### 5.3.1 Preclinical evidence

A first key unresolved issue is to determine whether the classification of individualized interventions as research or care should influence the type and amount of preclinical evidence required for approval, and if so, in what ways. Currently, there is relatively little guidance on the matter, with the only FDA guidance for nonclinical testing being for individualized antisense oligonucleotides (53) and none publicly available for personalized CRISPR/Cas9-based interventions. Challenges have been reported with determining suitable and validated measures, particularly for certain genetic heterogeneous conditions where outcome measures tend to be subjective (54). We argue that, while a threshold of evidence and methodological rigor should exist, the focus of regulatory scrutiny may need to be adjusted based on the overarching goals of the intervention and the specifics of each case.

As described earlier, if the primary/only goal of the intervention is to directly benefit the patient, then translational distance might be minimized by closely mimicking the patient’s specific circumstances in the preclinical study. If this is the case, assessment of preclinical evidence should be focused on patient-specific evidence. In the context of DMD, this might mean prioritizing *in vitro* evaluation of the efficiency of the CRISPR system in patient-derived muscle cells, assessing the similarities in genomic architecture between animal model and patient genome, or confirming that patient-specific characteristics are replicated in the preclinical study (age, co-interventions, etc). In other words, the emphasis might be placed on evaluating translational distance, including the following factors: (1) humanization of animal models (incorporation of human exons, entire genes, or organs) and implications for CRISPR/Cas9 off-target effects, including mitigation strategies, (2) similarities between patient and model’s immune systems, (3) similarities between experimental conditions in preclinical and clinical studies. For a detailed explanation of how to evaluate translational distance, see Kimmelman (5).^8^

Conversely, if one of the goals is to test a more broadly applicable intervention ensemble, the validity and generalizability of the proposed treatment algorithm should also be assessed. For example, in the case of DMD, an intervention ensemble that combines gene therapy and standard of care treatment might be tested, in the hope of better recapitulating the average patient’s situation in the clinic. Evaluation of the ensemble might involve judging whether the most widely applicable standard of care was used (e.g., glucocorticoids), in combination with the correct dosage and dosing schedule (e.g., starting around age 4–5 in humans, given daily or weekly) and assessments of the validity of equivalencies made between animal models and humans (e.g., regarding age or immune system). Of course, in both cases, patient-specific evidence and evidence related to the treatment algorithm may be assessed but the key decision lies in which type of evidence should take precedence based on the intervention’s intended goals.

#### 5.3.2 Data collection/clinical trial design

Another feature typically evaluated by regulatory agencies is the proposed clinical trial design, or the way the intervention is intended to be administered to patients. Here again, several parameters can be discussed and adjusted based on the specifics of each case. A key aspect that may vary depending on the specific patient and intervention is the type and amount of data collected during the trial. As of now, little guidance exists on what type of data should be collected during n-of-1 trials and data collected as part of expanded access programs - another setting where research and care are intertwined - seem to be relatively unhelpful, as noted by Chapman et al. (17).^9^ We hope our model facilitates a proactive and informed decision on this matter, ensuring ongoing alignment between the preferences and values of all partners.

The choice of data collection methods should reflect both the primary objectives of the intervention and the individual patient’s needs and preferences. This includes finding an appropriate balance between generating valuable data and minimizing the logistical burden on the patient and their family and associated costs (17, 71).

First, the objectives of the intervention may dictate whether a control is necessary. For individualized interventions, controls can take the form of data collected on the patient prior to the intervention or high-quality natural history data (68). An intervention that is administered solely under a therapeutic warrant may not require such robust controls, whereas one that has research purposes will. Moreover, if the primary focus is on patient benefits, the data collected may be limited to what is strictly necessary for enhancing care and assessing patient outcomes. Conversely, if there is a consensus that the intervention may be designed to also contribute to broader societal benefits, then a more comprehensive data collection approach must be employed.

Going back to our case study, consider Patient B (10 years old, AAV9) and Patient C (10 years old, MyoAAV). Consider that all parties involved in the case of Patient B initially agreed that access to therapy should be prioritized, therefore intentionally limiting research aspects. Consequently, data collection might be minimal, perhaps only evaluating parameters such as expression of the transgene/dystrophin and the North Star Ambulatory Assessment (NSAA), a 17-items measurement tool commonly used in DMD trials (55).^10^ On the other hand, let us assume it was agreed that the intervention for Patient C would only be justified if one of the goals was to generate knowledge on the use of the novel MyoAAV in humans. In this case, a more extensive data collection strategy would be implemented. This might range from incorporating a more detailed assessment of immune reactions and toxicity, to a greater number of data collection timepoints to capture the effects of the intervention over time, to an evaluation of specific biomarkers with the aim of testing novel hypotheses. These considerations would need to be built-in to the trial design from the outset.

Beyond tailoring the extent of clinical data collection to each specific case, clinical endpoints should also be adapted accordingly. Currently, little guidance exists on which clinical endpoints should be utilized in n-of-1 trials (17). Traditional primary endpoints may require years to establish efficacy, and surrogate endpoints, though faster to assess, may not reliably indicate effectiveness in the intended patient population. As novel treatments are developed for individual patients, there may be a need for establishing novel types of endpoints to confirm efficacy. Although novel and/or tailored endpoints have the potential to facilitate early identification of treatment efficacy or adverse events, they come with significant drawbacks. A major concern is the potential lack of robust validation, which could take years to establish and, in the meantime, cast doubts on the reliability of results obtained through their use. Establishing meaningful and reliable endpoints is crucial in the informed consent process and for both clinical and research purposes. Moreover, novel endpoints intended to demonstrate efficacy in one patient may not be broadly applicable or generalizable to a wider patient population, limiting their utility. These considerations highlight the need to develop guidelines for data collection (including clinical endpoints) for individualized interventions, possibly in a disease-specific or technology-specific manner. A balance must be struck here, as developing disease-specific or technology-specific guidelines risks deviating from the tailored regulatory approach we advocate.

#### 5.3.3 Data generalizability and standardization

When research endeavors are both permitted and desired, regulatory agencies can also review the extent to which generalizability and standardization are built-in to the trial, therefore ensuring alignment between the goals of the intervention and the proposed trial design. As discussed above, ensuring generalizability may involve broadening translational distance to an appropriate level to optimize potential benefits for a more diverse patient population.

Ensuring generalizability may also mean selecting clinical outcomes that can easily be aggregated across different studies. On the other hand, if the goal of the intervention is primarily therapeutic, only clinical outcomes that are relevant to the patient’s specific case, whether or not they can easily be aggregated, may be chosen. When there is an intention to aggregate results from several studies to enhance data generalizability, a thorough evaluation of data harmonization practices may be required by regulatory agencies. This may include standardizing elements such as standards of care, the use of Electronic Health Records, data collection methods, nature and length of patient follow-ups and data reporting in disease registries (56). Clinical data harmonization and aggregation have historically posed significant challenges across various fields (57, 58), and individualized interventions are no exception. While it may be overly ambitious to standardize all n-of-1 trials, developing disease-specific or technology-specific guidelines could be a practical first step toward standardizing n-of-1 trials that target the same condition or utilize the same technology. For example, in the case of DMD, a subset of key endpoints, such as expression of the transgene/dystrophin, and NSAA, could be identified as a minimum requirement, allowing for consistent comparisons of these critical metrics across different studies. Establishing these principles may enhance the reliability and comparability of individualized interventions and facilitate their aggregation and analysis. Knowing which studies to aggregate will present a challenge given the diversity in patient populations, delivery methodS/vectorS and therapeutic systemS. Researchers and regulatory bodies will need to work together and use judgment and mechanistic reasoning to determine the similarities and differences between diverse populations, interventions and systems and determine if two n-of-1 studies are “sufficiently” similar to be aggregated.

Standardizing studies that utilize the same viral vector or gene editing technology for a specific disease could also facilitate the evaluation of their safety and efficacy (5, ch. 4), potentially streamlining approval processes of similar systems for future patients. For example, in the case of DMD, once a certain number of personalized CRISPR-based interventions have been tested for different patients and mutations but for the same mutation type (eg., duplications) and using the same vector (e.g., MyoAAV), will the FDA require new animal models to be generated for each new patient? Or would the data collected for previous patients, showing efficacy of the overarching gene editing approach, and *in vitro* preclinical data testing the personalized system in the individual patient’s cells be sufficient? If the regulatory oversight can indeed be streamlined for subsequent patients, how many times would the FDA need to see data in personalized animal models first? Our model recognizes that, while we may not be there yet, certain individualized interventions could eventually be considered a purely therapeutic endeavor, particularly if only specific elements of the therapeutic platform are modified for each new patient. Additional guidance is required to determine when these interventions would transition to being considered solely therapeutic.

Another step that can be taken toward standardizing n-of-1 trials with a research objective, and which could also address justice concerns, is to administer individualized interventions at specialized care centers. This would ensure comprehensive monitoring and treatment of all healthcare needs and potential adverse events. However, administering bespoke therapies through specialized centers may burden families and patients with significant travel and accommodation expenses. In some cases, patients and their families may even need to relocate if the trial sites are situated in a different country. Additional obstacles may include taking extended time off work for family members and covering costs associated with caring for other children. While we recognize that equitable access is a deeply complex issue, institutions should actively seek solutions to address the specific barriers outlined above. Providing a dedicated contact person to assist with travel and accommodation arrangements could be beneficial, and all travel-related costs should be borne by the trial team.

In this section, we show that several parameters can be adjusted based on the specifics of each case: (1) the focus of regulatory review regarding preclinical evidence, emphasizing patient-specific evidence or evidence related to the validity of the intervention algorithm, (2) the scope and nature of data collected during the intervention, and (3) the extent to which the trial incorporates generalizability and data standardization practices. Categorizing individualized interventions as leaning toward either research or care may also change the way clinical success is defined. For example, an intervention that is primarily justified based on a therapeutic warrant may define success in terms of patient-centered outcomes, such as health improvements or quality of life. On the other hand, in an intervention that is designed to enhance knowledge gains and societal benefits, success may be defined, at least in part, based on the amount and quality of knowledge gained through the trial.

### 5.4 Step 4: post-intervention evaluation and follow-up

The process of ethically and safely delivering individualized interventions goes beyond the administration of the intervention and requires thorough evaluation of the data collected during the trial as well as continued patient follow-up to monitor safety and efficacy. The current lack of clarity in distinguishing individualized interventions as either research or care has compounded pre-existing difficulties in evaluating and reporting n-of-1 trials.

As noted by Shamseer et al. (59), there is substantial evidence of incomplete, inaccurate, and opaque reporting of n-of-1 studies. For example, only 3% of all published n-of-1 studies appear to have a prospectively registered protocol (8) and fewer than half provided enough information for meta-analysis, including 79% of studies failing to indicate their primary outcome (59).

To mitigate these issues, several guidelines, including the CONSORT/CENT and SPIRIT/SPENT guidelines, outline best practices for reporting n-of-1 interventions that primarily have a research objective (59, 60). However, these guidelines seem to be primarily focused on crossover trial designs, as evidenced by their emphasis on terms such as “randomization” (Item 1a), “washout periods” (Item 3a) and “blinding” (Item 11a), thus leaving many questions unresolved for the reporting of CRISPR/Cas9-based interventions that necessitate a pre-post trial design. However, the core principle of these guidelines—that reporting individualized interventions be more transparent and detailed—should certainly also be applied to pre-post n-of-1 trials.

Beyond the general need for rigorous data evaluation, interpretation and transparent reporting, we believe that this phase should, once again, be tailored to the specifics of each patient case and intervention. Key aspects that may be adapted include: (1) the extent and nature of data reporting, and (2) the duration and scope of patient follow-up.

#### 5.4.1 Data evaluation and reporting

First, the extent and nature of data reporting as well as data sharing practices may need to be adjusted based on the specific goals of the intervention. We argue that the primary objectives of any intervention should be clearly articulated in any reporting outlet used. When it comes to individual cases, data reporting for interventions aimed at therapeutic outcomes might be restricted to the patient’s clinical results, primarily derived from medical records and patient-reported outcomes. In this case, data sharing might be limited to the patient’s healthcare team and the administering institution, particularly if the data is unlikely to benefit other patients.

On the other hand, if one of the goals of the intervention was to generate generalizable knowledge, data reporting might be more comprehensive and emphasize statistical analysis and the potential for aggregation with other (current or future) trial data. This includes a focus on reproducibility and detailed methodological descriptions, which may not be as critical for interventions that only have a therapeutic goal. Interventions designed to maximize knowledge gains and societal benefits may require broader data sharing, including submissions to peer-reviewed journals, regulatory agencies, and the wider scientific and medical communities. Here again, our model is designed to identify these key decisions early on and distribute responsibility across multiple partners and institutions.

If there is an intention to evaluate and pool results across several n-of-1 studies, this should be clearly indicated in the reporting of the study results. The CENT guidelines offer clear recommendations for reporting data from a series of n-of-1 trials (59) and recommends clearly distinguishing between elements of design that are standardized across studies and those that are individualized for each patient/trial (59). They also provide guidance on how to report statistical analyses for aggregated n-of-1 trials, including the use of Bayesian techniques (Zucker et al., 2010). These considerations should be integrated into trial design and reported transparently when data from multiple trials are aggregated. In addition to developing data harmonization practices, incentives should also be established to encourage institutions to share data with all parties involved, as financial and legal challenges have often hindered such collaboration.

#### 5.4.2 Follow-up

Although the FDA published draft guidance for IND submissions for some n-of-1 interventions, guidance and recommendations for patient follow-up remain vague (61). In larger gene therapy trials, the length of follow-up recommended by the European Medical Agency and the FDA range between 5 and 15 years (62). Once again, the duration (and nature) of patient follow-up should be tailored to each intervention, with more extensive monitoring needed for interventions that are aimed at generating broader knowledge. Practical barriers to successful follow-up should be considered by researchers and institutions providing bespoke interventions, including the costs associated with travel to medical centres. To mitigate these challenges, adopting a decentralized strategy is advisable as it enables assessments to occur at local facilities whenever possible. This approach helps ease the challenges faced by patients and caregivers while ensuring the ongoing collection of safety and efficacy data. Additionally, data from both successful and halted trials should be collected in a standardized format and made publicly available (5, 68).

## 6 Justice considerations

The last four sections of this paper introduced a conceptual model as well as practical insights for developing, administering and evaluating individualized interventions. When it comes to implementing this model into practice, institutions should keep justice concerns at the forefront of all decision-making, ensuring such research is accessible to a diverse range of participants.

In the rare disease space, large patient advocacy groups are better positioned to advocate for and fund research into innovative interventions. However, there are thousands of rare diseases that have high unmet need, all deserving of research opportunities. Institutions must weigh the cost of developing programs alongside chances of successful outcomes while also balancing opportunities for underserved or underrepresented patient populations.

At times, families of patients may raise funds on their own to help initiate a trial for their loved one. They may do this due to the absence of advocacy groups raising funds or promoting research on their child’s condition, or because they believe pre-existing research projects will not have a meaningful impact on themselves or their child.

In certain cases, patient-funded trials have a negative impact on solidarity within a patient community. Family-initiated and -funded individualized research automatically excludes others within the patient population that might be served by similar research. Parents taking on the burden of funding research for their own children may inadvertently create an unintended equity gap between those who can and those who cannot afford the high costs associated with individualized interventions. Some families may fundraise to pay for access through crowd-sourcing and social media campaigns. Though understandable, these campaigns typically create further divide within patient communities, as some families are computer savvy, have well-connected social networks, and are skilled communicators, while others are not. These initiatives frequently benefit the former group over the latter, and successful campaigns are generally based on perceived social worth rather than genuine medical necessity.

Some families without the ability to contribute financially to research endeavors may also feel ostracized within their community and excluded from research opportunities as a result. Additionally, there could be a perception of influence for families that have successfully launched research, with institutions giving more weight to perspectives from parent-funders. Power imbalances and competing priorities within patient communities may arise, undermining the shared mission to find curative approaches for the entire patient population. Further divides may be created if parents are able to raise capital but want researchers to prioritize specific therapeutic approaches or create interventions designed to impact different aspects of the disease progression.

To address these potential negative impacts on justice for patients, it is crucial to carefully consider inclusivity, transparency, and fair participation in patient-funded trials to ensure that every member of the patient community feels respected, involved, and empowered. All parties involved in developing customized treatments should work toward equal access for all, regardless of their socioeconomic status, location, or other demographic characteristics. The design and execution of personalized trials should emphasize inclusivity and strive to address discrepancies in healthcare access to prevent marginalized populations from being disproportionately disadvantaged. By proactively tackling issues of fairness, personalized trials can aim to advance just, diverse, and socially equitable practices in the field of precision medicine.

## 7 Next steps: how to evaluate our model?

As noted earlier, further discussions among partners are necessary to determine how our new model and practical guidance can be incorporated into existing frameworks and evolving regulations, or whether new pathways need to be established. Once integrated into practice, the effectiveness of our model will need to be demonstrated using tangible metrics and rigorous evidence. It is important to consider the types of evidence required to validate that this model effectively meets both individual and societal needs.

To evaluate the effectiveness and adequacy of our new model, the following approaches may be considered:

### 7.1 Case study analysis

Our new model could be implemented in a real-world scenario, such as an individualized CRISPR/Cas9-based intervention for a patient with Duchenne Muscular Dystrophy. If possible, outcomes should be compared to other cases that were developed, administered, and evaluated using more traditional approaches and pathways.

### 7.2 Partner feedback

Importantly, partner feedback, including insights from patients, families, clinicians, researchers, and regulatory bodies, should be gathered to assess their experiences using the model, focusing on aspects like clarity, flexibility, transparency and alignment with patient needs and overall objectives. We anticipate that our model could benefit patients and families - by ensuring their needs and preferences are more fully addressed - and researchers - by distributing the responsibility of decision-making across various partners. However, only those directly involved can provide meaningful insights into the model’s practical impact.

### 7.3 Adaptability

Considering its emphasis on tailoring the goals of an intervention to the specifics of each case, it appears essential to evaluate the model’s flexibility in accommodating diverse patient cases and interventions, considering whether it can be easily adjusted to suit different contexts.

Evaluating models for the development and evaluation of individualized therapies can be challenging due to varying definitions of success among partners and the presence of intangible or difficult-to-measure factors. However, metrics developed by Lynch et al. (63) to assess institutional review board (IRB) quality may offer a useful framework here. These metrics include:

- *Efficiency*: Measured by factors such as turnaround time.
- *Facilitation of research*: Assessed through researcher satisfaction surveys and clarity of communication.
- *Compliance*: Adherence to regulatory and institutional policies.
- *Ethical principles*: Autonomy, justice, beneficence and transparency evaluated through auditing and post-approval monitoring.
- *Quality of review*: Transparent, consistent, and thoughtful decision-making over time.
- *Participant protection*: Measured by participant understanding of the consent process, participant satisfaction and experience, and the number and severity of adverse events.

For a more detailed exploration of these metrics, see Lynch et al. (64).

## 8 Discussion

The question of whether individualized interventions should be categorized primarily as research or care has significant implications for institutions involved in their development, administration and evaluation. Traditionally, this classification has influenced various aspects of patient management, from the initial planning and development stages to post-intervention evaluation and follow-up. However, navigating this continuum can be complex, as little guidance is provided on the issue. Here, we introduce a new conceptual model as well as practical insights for developing, administering, and evaluating individualized interventions, with a focus on CRISPR/Cas9-based interventions.

This new model provides a structured approach to addressing the complexities inherent to individualized interventions and offers a framework for understanding and managing the interplay between research objectives and patient care needs. Our model rests on three central principles: (1) it emphasizes that the goals and design of the intervention should be as personalized as the bespoke product itself; (2) it suggests that, while the research-care categorization has limited utility at a higher (i.e., whole intervention) level, it can, and should, be used to make assessments at the level of each individual component of the intervention, therefore focusing on the practical details of how such interventions are administered; and (3) it promotes open dialogue among all partners to anticipate and adjust various parameters to suit the unique needs of each case and address ethical tensions raised by bespoke therapies involving informed consent and risk/benefit analysis.

Importantly, in this paper, we do not seek to provide a definitive answer to where individualized interventions in general fall on the research-care continuum as we recognize that there is no one-size-fits-all approach for all bespoke therapies. Though our model could be used to locate individual trials on this continuum, our primary aim is the assessment of the component parts of individualized interventions and the location on the research-care continuum of parameters associated with these components. These key parameters are found in Table 1. Concretely, our hope is that this model will provide operational guidance concerning the development, administration and evaluation of individualized interventions based on the individualized goals of each trial.

**Table 1:** Adjustable parameters for the development, regulatory oversight and evaluation of individualized interventions.

Step	Adjustable parameter
Planning and development	Minimizing translational distance vs. testing a more broadly applicable treatment algorithm
	Investigating broader scientific questions vs. ensuring timely patient access to therapy
Regulatory oversight	Amount and type of preclinical evidence required for approval
	Amount and type of data collected during the intervention
	Built-in data generalizability and standardization
Post-intervention evaluation	Extent and type of data sharing and reporting
	Length and type of patient follow-up

### 8.1 Conclusion

Below, we summarize the key implications of our model:

#### 8.1.1 Initial discussions

Our model emphasizes the importance of early, transparent discussions among all partners to come to an agreement on the overarching goals of the intervention. This helps in setting clear and transparent priorities and expectations from the outset, ensuring that all perspectives are considered and integrated into the decision-making process, thereby minimizing therapeutic misconception and overestimation. Further work is required to understand how these conversations could be facilitated and determine the relative weight of each partner in the decision process.

#### 8.1.2 Planning and development

Our model highlights the importance of balancing the need to generate novel, generalizable knowledge in laboratory settings and ensure timely patient access to the therapy. This decision dictates how time and resources may be allocated and the degree to which translational distance should be minimized, or alternatively, broadened, to assess the applicability of a more general treatment algorithm. Importantly, our model ensures the responsibility for decision-making is distributed among all parties involved and ongoing alignment between the preferences and values of all partners is maintained.

#### 8.1.3 Regulatory oversight

Our model suggests that the nature and extent of preclinical evidence required by regulatory agencies, the type of data collected during the trial, and the degree of data generalizability and standardization built-in to the trial should be tailored based on the intervention’s primary objectives and patient-specific circumstances. Developing disease-specific or technology-specific guidelines could be a practical step toward standardizing n-of-1 trials that target the same condition or utilize the same technology.

#### 8.1.4 Post-intervention evaluation and monitoring

While guidelines are continually evolving to address challenges related to the rigorous and transparent evaluation and reporting of n-of-1 trials generally, our model suggests that the extent of data reporting and data sharing practices may also need to be adjusted based on the primary goals of the interventions. The duration and nature of patient follow-up should also be tailored to the specifics of each case.

### 8.2 Unanswered questions and limitations

Regulatory bodies as well as scholars are continuously working toward updating guidelines and adjusting existing regulatory pathways to address the novel and unique ethical challenges that are raised by the rapid pace of development of individualized interventions. There remain significant unanswered questions, such as which patients should be given priority access to a bespoke gene therapy, how institutions should handle equitable access to individualized interventions, or how advocacy groups contribute to reshaping the boundary between research and care, some of which will be explored further in upcoming papers. Discussions will be essential to define the precise role of IRBs and to assess how variations in their governance practices may affect the implementation of our proposed model.

We hope this paper contributes to ongoing discussions around determining where personalized interventions fall on the research-care continuum and understanding the institutional, regulatory, and ethical implications of classifying custom therapies as research, care, or a mix of both. Whether our new model and recommendations can be integrated into existing pathways or whether new processes may need to be developed remains open for discussion. We look forward to engaging with diverse partners to collaboratively explore the most effective path forward.

We close by noting limitations and work yet to be done. Though we have described in outline a new model for the assessment of individualized interventions, we have not yet provided an exhaustive analysis of the parameters involved, nor provided explicit ethical guidance regarding how these parameters ought to be assessed. Another significant challenge that we have not addressed in this paper is the implications of this model for regulation and oversight. We hope to pursue this more detailed work in the future.

## Data Availability

The original contributions presented in the study are included in the article/supplementary material, further inquiries can be directed to the corresponding author.

## Footnotes

^1^To illustrate the research-care continuum, Crowden et al. (7) give the following example: “A clinician wishes to monitor the extent to which she is successful in achieving full vaccination for all children in her practice. No one is likely to suggest that she is conducting research, or that she had better appear before an institutional review board or risk subsequent censure from her colleagues when her clandestine research activities are brought to light. What if she wishes to conduct her monitoring in collaboration with a number of colleagues with one goal: to ultimately compare how well each of them is doing? What if, as a group, these physicians negotiate with the local public health department for a public health nurse to help them establish registries of their patients with systematic reminders to help achieve full vaccination? What if they now monitor, in a before-after fashion, the extent to which the intervention of the health department improved the vaccination rate? Finally, what if the group decides to publish the results of their experience, in the hopes that they might be beneficial to others? At what point in this series of possible activities related to vaccination does the transition from quality assurance not requiring research ethics oversight to a research activity requiring such oversight occur?” (p. 129).

^2^We recommend looking at (8) for a complete history of n-of-1 trials.

^3^Kane et al. (10) give the example of a trial evaluating the use of CAR-T cells in children with leukemia, where the intervention was administered under the categorization of innovative care all the while promoting data collection, therefore not requiring conventional research oversight.

^4^This mirrors, in certain ways, Weijer’s component analysis for the ethical assessment of risks in research and clinical setting (70).

^5^For further discussion of the advantages and risks across different patient populations, including considerations around age and disease stage, we recommend reading Iyer et al. (50).

^6^Providing and gaining informed consent is one of the guiding ethical principles of human research. The Belmont Report is an influential publication outlining ethical guidelines for ensuring that the informed consent process is robust. In Canada, the Tri-Council Policy Statement regarding ethical conduct for research involving humans (TCPS 2) echoes the Belmont Report and requires participants to have all the information available to researchers regarding safety and efficacy of interventions prior to agreeing to participate in a clinical trial.

^7^Johnston et al. (32) note that this was the case for the recent Milasen case: “The oversight and regulatory approaches applied to Milasen were a complex and alternating mix of human subjects research and clinical innovation processes. To develop the ASO, researchers needed to collect blood and skin cells from Mila. This aspect was understood as human subjects research, overseen by the Boston Children’s Institutional Review Board (IRB). Yet, at the point of administration, Milasen appears to have been treated as a clinical intervention, although a novel one. Although Mila’s doctors obtained the concurrence of the IRB chair, Mila received the intervention under an expanded access approval from the FDA, an exception to usual FDA regulations for patients not eligible for an ongoing clinical trial. Access in these cases is driven by clinical need, and patients under an expanded use approval are not research subjects. But in the Milasen case, there was no clinical trial—the single expanded access patient was the only person receiving the intervention, a fact that was clear to the FDA.” (p. 2).

^8^We acknowledge that we may be using the concept of “translational distance” in a slightly different way than Kimmelman does (5). Based on how we conceptualize it here, translational distance would necessarily increase as we move from an individualized intervention to a generalizable approach. Kimmelman notes, and we agree, that there are additional important considerations to assess translational distance and associated risks, such as the quality of preclinical evidence, novelty of the platform, accuracy of animal models, etc.

^9^Chapman et al. (17) write the following: “FDA draft guidance recommends long-term follow-up observations on patients receiving gene therapy products unless the risk of delayed adverse events is low. In April 2018, a former FDA official said that the agency has traditionally not viewed data collected in EA [Expanded Access] settings as useful, but added that if companies “move to a more protocol-driven environment for expanded access, where we could do a much better job of getting reliable data, then I think we might be able to change this concern.” Still, there are reasons for caution. If EA or RTT is viewed more like research than treatment, and involves more than minimal data collection and thus increases the administrative burden on physicians, its primary purpose of providing timely access to investigational products to patients with no other therapeutic options may be threatened. The FDA should issue guidance to clarify this issue for all investigational drugs and biologics, including gene therapies.” (p. 353).

^10^These endpoints, as well as secondary endpoints, such as time to rise from the floor, four stair climb and 10 min walk-run, were used in the ELEVIDYS trials (65). ELEVIDYS is an AAV-based gene therapy which encodes a micro-dystrophin protein that contains the essential domains of dystrophin. The expression of micro-dystrophin as well as secondary endpoints were instrumental in the FDA decision to extend approval for DMD patients aged 4 years and older (instead of 4–5 years as was granted in the first approval) (66). Of important note, approval of this treatment raises several important questions such as: which primary and secondary outcomes should be used to best evaluate efficacy? Can approval be granted if secondary (or exploratory) endpoints show significant improvements but the study failed to meet its primary endpoints?
